# CD38 in the nucleus accumbens and oxytocin are related to paternal behavior in mice

**DOI:** 10.1186/1756-6606-6-41

**Published:** 2013-09-23

**Authors:** Shirin Akther, Natalia Korshnova, Jing Zhong, Mingkun Liang, Stanislav M Cherepanov, Olga Lopatina, Yulia K Komleva, Alla B Salmina, Tomoko Nishimura, Azam AKM Fakhrul, Hirokazu Hirai, Ichiro Kato, Yasuhiko Yamamoto, Shin Takasawa, Hiroshi Okamoto, Haruhiro Higashida

**Affiliations:** 1 Kanazawa University Center for Child Mental Development, Kanazawa 920-8640, Japan; 2 Department of Biophysical Genetics, Kanazawa University Graduate School of Medical Sciences, Kanazawa 920-8640, Japan; 3 Department of Biochemistry, Medical, Pharmaceutical and Toxicological Chemistry, Krasnoyarsk State Medical University, Krasnoyarsk 660022, Russia; 4 Department of Neurophysiology, Gunma University Graduate School of Medicine, Maebashi, Gunma 371-8511, Japan; 5 Department of Biochemistry, Graduate School of Medicine and Pharmaceutical Sciences, University of Toyama, Toyama 930-0194, Japan; 6 Department of Biochemistry and Molecular Vascular Biology, Kanazawa University Graduate School of Medical Sciences, Kanazawa 920-8640, Japan; 7 Department of Biochemistry, Nara Medical University, Kashihara 634-8521, Japan; 8 Department of Advanced Biological Sciences for Regeneration, Tohoku University Graduate School of Medicine, Sendai 980-8575, Japan

**Keywords:** Parental behavior, Paternal behavior, Maternal behavior, Retrieval behavior, Oxytocin, CD38, Nucleus accumbens

## Abstract

**Background:**

Mammalian sires participate in infant care. We previously demonstrated that sires of a strain of nonmonogamous laboratory mice initiate parental retrieval behavior in response to olfactory and auditory signals from the dam during isolation in a new environment. This behavior is rapidly lost in the absence of such signals when the sires are caged alone. The neural circuitry and hormones that control paternal behavior are not well-understood. CD38, a membrane glycoprotein, catalyzes synthesis of cyclic ADP-ribose and facilitates oxytocin (OT) secretion due to cyclic ADP-ribose-dependent increases in cytosolic free calcium concentrations in oxytocinergic neurons in the hypothalamus. In this paper, we studied CD38 in the nucleus accumbens (NAcc) and the role of OT on paternal pup retrieval behavior using CD38 knockout (CD38^−/−^) mice of the ICR strain.

**Results:**

CD38^−/−^ sires failed to retrieve when they were reunited with their pups after isolation together with the mate dams, but not with pup, in a novel cage for 10 min. CD38^−/−^ sires treated with a single subcutaneous injection of OT exhibited recovery in the retrieval events when caged with CD38^−/−^ dams treated with OT. We introduced human CD38 in the NAcc of CD38^−/−^ sires using a lentiviral infection technique and examined the effects of local expression of CD38. Pairs of knockout dams treated with OT and sires expressing CD38 in the NAcc showed more retrieval (83% of wild-type sire levels). Complete recovery of retrieval was obtained in sires with the expression of CD38 in the NAcc in combination with OT administration. Other paternal behaviors, including pup grooming, crouching and huddling, were also more common in CD38^−/−^ sires with CD38 expression in the NAcc compared with those in CD38^−/−^ sires without CD38 expression in the NAcc.

**Conclusions:**

CD38 in the NAcc and OT are critical in paternal behavior.

## Background

The survival of mammalian young is dependent on assistance by parents or alloparents [1]. In all mammalian species, becoming a mother involves remarkable behavioral changes that are driven by a combination of neuroendocrine and experiential factors [2-6]. Maternal behavior is defined as the repertoire of a mother’s behaviors that increase offspring survival [1,4]. Similar nurturing behavior by the male parent is also important to newborns [7-10].

Most mice, rats and hamsters are not monogamous and males are not spontaneously maternal [8,11-15]. However, if male and female mice are forced to live together for long periods in a small cage, the male mice will inevitably be found in the nest with offspring, providing protection and warmth. This has been described as the “sensitization process” in male rats [16].

We have previously demonstrated that nonmonogamous ICR mouse sires initiated maternal-like parental care (retrieval of pups, pup grooming and crouching) when they were housed continuously with their mates and pups [17,18]. Under stressful conditions, such as isolation alone in a new environment (cage) for 5–10 min, the sires did not show retrieval when they were reunited with pups. These same sires had previously shown paternal behavior for 3 or more days after the pups’ birth. The sire retrieved the pups if they were caged together with his mate (the pups’ dam). The dam emitted unique 38-kHz ultrasonic vocalizations (USVs) to their mate (the pups’ sire) during this period. These 38-kHz USV signals and unidentified pheromones from the maternal mate caused the paternal retrieval behavior [17].

It has been demonstrated that the medial preoptic area (mPOA), ventral tegmental area (VTA), nucleus accumbens (NAcc) and ventral pallidum (VP) can interact in the regulation of maternal behavior in rodent females [19-24]. Briefly, mPOA neurons activate VTA dopaminergic neurons that innervate GABA neurons with D1 receptors in the NAcc. GABAergic neurons of the NAcc inhibit VP neurons that trigger maternal behavior in dams. Oxytocin (OT) receptors are also found in the NAcc of spontaneously maternal females [24]. Oxytocinergic modulation from the mPOA affects VTA and NAcc neurons. Hypothalamic oxytocinergic neurons directly innervate the NAcc [22-26]. Although such neurocircuits have been proposed to be involved in female maternal behavior, we hypothesized that OT also functioned as a transmitter or neuromodulator in the NAcc of males showing parental behavior.

OT is synthesized in neurons in the paraventricular nucleus and supraoptic nucleus of the hypothalamus, is secreted somato-dendritically from OT-containing neurons into the brain, and causes excitation by activating OT receptors on oxytocinergic neurons [27-31]. This hormone plays a critical role in social recognition and behavior in mammals [28-35]. It has been previously demonstrated that CD38 [36-39], a type II transmembrane protein, catalyzes the genesis of the second messenger cyclic ADP-ribose for Ca^2+^ mobilization in glucose-induced insulin secretion [36]. The similar mechanism has been found in other physiologically important processes: CD38 is strongly expressed in the hypothalamus and is crucial for the release of OT from oxytocinergic neurons but not for vasopressin secretion in the mouse hypothalamus [39]. Thereby, social memory and recognition in males and nurturing behavior in maternal females were disrupted in CD38 knockout mice by reduced OT secretion [31,32,39]. It has been reported that two single nucleotide polymorphisms (SNPs) of CD38 are associated with autism spectrum disorder (ASD) or at least represent a weak risk factor in ASD subjects in the U.S.A., Israel and Japan [40-45]: One of such CD38 SNPs has previously been detected as a risk factor in Japanese patients with diabetes [46,47].

Our unique paradigm of parental behavior [17] addresses the question of whether OT and CD38 in the NAcc are involved in paternal behavior. We used CD38 knockout (CD38^−/−^) mice [39,48] to assess this hypothesis. We examined retrieval in CD38^−/−^ mice with or without subcutaneous injection of OT. Next, we examined sires with or without local expression of human CD38 (HCD38) in the NAcc using a lentivirus infection method that had been described previously [39]. Finally, we histologically confirmed the expression of HCD38 and determined functional recovery by examining aversive behavior using the sucrose preference test. Sucrose intake is dependent on the reward circuits of the NAcc, and this reward is related to pup-dependent parental care [49-57].

## Results

Approximately 60% of the wild-type ICR sires displayed parental retrieval behavior (n = 30, Table 1, 1st row), as previously reported [17]. The CD38^−/−^ sires failed to retrieve their pups after separation from their CD38^−/−^ mate dams in a novel cage for 10 min (n = 30, Table 1, 2nd row). This finding was confirmed by comparison with a sample of wild-type or knockout sires and dams (Table 1, 3rd and 4th rows).

**Table 1:** **Parental behavior in sires after pairing in different combinations of wild-type (CD38**^**+/+**^**) and CD38**^**−/− **^**mice with or without an oxytocin (OT) injection**

**Female**	**Male**	**Percentage of male’s exhibiting retrieval behavior**		
		**(n)**	***P *value (1)**	***P *value (2)**
CD38^+/+^	CD38^+/+^	60 (30)		
CD38^−/−^	CD38^−/−^	0 (30)	*P* = 0.0000	
CD38^+/+^	CD38^−/−^	0 (10)	*P* = 0.0000	
CD38^−/−^	CD38^+/+^	0 (10)	*P* = 0.0000	
CD38^−/−^ +OT	CD38^−/−^	0 (8)	*P* = 0.0035	
CD38^−/−^	CD38^−/−^ + OT	0 (8)	*P* = 0.0035	
CD38^−/−^ +OT	CD38^−/−^ + OT	30 (30)	*P* = 0.0370	*P* = 0.0019

*P* value (1) represents two-tailed *P* values by Fisher’s exact test between the first row (the CD38^+/+^/CD38^+/+^ pair) and each of the other rows. *P* value (2) represents two-tailed *P* value by Fisher’s exact test between the second (the CD38^−/−^/CD38^−/−^ pair) and seventh row (the CD38^−/−^ +OT/CD38^−/−^ +OT pair).

Pairs with a single subcutaneous injection of OT at a concentration of 100 ng/kg body weight to either the CD38^−/−^ sire or dam did not initiate parental retrieval behavior (Table 1, 5th and 6th rows). However, 30% of the OT-treated CD38^−/−^ sires that were co-housed with the OT-treated dams showed retrieval behavior (n = 30, *P* = 0.0370 from the CD38^+/+^/CD38^+/+^ pair; *P* = 0.0019 from the CD38^−/−^/CD38^−/−^ pair; Table 1, 7th row; Fisher’s exact test), This frequency is 50% of that shown by untreated wild-type sires caged with untreated wild-type dams.

Expression of GFP as a control or the re-expression of HCD38 in the NAcc in mating males was performed via lentiviral infection, as has been reported previously [39]. The retrieval behavior of the sires was scored more than 2 weeks after recovery from surgery and then siring pups by the untreated mate dams. Pairs consisting of sires expressing GFP and knockout dams treated with OT (Table 2, 2nd row) or without OT (Table 2, 1st row) did not exhibit retrieval. Thirty percent of the GFP-sires that were treated with OT showed retrieval when they were co-housed for 10 min in a new environment with OT-treated CD38^−/−^ dams, (n = 10; Table 2, 3rd row). This frequency of retrieval is 50% of that shown by the wild-type sires.

**Table 2:** **Parental behavior by sires after pairing in different combinations of CD38**^**−/− **^**mice transfected with either GFP or HCD38 in the NAcc with or without an OT injection**

**Female**	**Male**	**Percentage of male’s exhibiting retrieval behavior**			
		**(n)**	***P *value (1)**	**(2)**	**(3)**
CD38^−/−^	CD38^−/−^ +GFP	0 (5)			
CD38^−/−^ +OT	CD38^−/−^ +GFP	0 (8)			
CD38^−/−^ +OT	CD38^−/−^+GFP+OT	30 (10)	*P* = 0.5055		
CD38^−/−^	CD38^−/−^ +HCD38	0 (10)			
CD38^−/−^ +OT	CD38^−/−^ +HCD38	50 (10)	*P* = 0.1090	*P* = 0.6499	
CD38^−/−^ +OT	CD38^−/−^ +HCD38+OT	60 (10)	*P* = 0.0440	*P* =0.3698	*P* = 1.00

*P* value (1) represents two-tailed *P* values by Fisher’s exact test between the first row (the CD38^−/−^/CD38^−/−^ + GFP pair) and the third, fifth, and sixth row. *P* value (2) represents two-tailed *P* value by Fisher’s exact test between the third (the CD38^−/−^ OT/CD38^−/−^ +GFP + OT pair) and the fifth and sixth row. *P* value (3) represents the value between the fifth and sixth row.

The CD38^−/−^ sires expressing HCD38 in the NAcc did not display retrieval when co-housed with the CD38^−/−^ dams (Table 2, 4th row). However, 50% of the HCD38 sires (83% of the level shown by the wild-type parents) showed retrieval when co-housed with the CD38^−/−^ dams that were treated with OT (Table 2, 5th row; n=10, *P* = 0.1090 compared with the CD38^*−/−*^/CD38^−/−^ + GFP pair; *P* = 0.6499 compared with the CD38^−/−^ + OT/CD38^−/−^ + GFP pair). Sixty percent of OT-treated sires expressing HCD38 showed full recovery of retrieval behaviors when they were co-housed with CD38^−/−^ dams with OT (Table 2, 6th row; n = 10, *P* = 0.0440 compared with the CD38^*−/−*^/CD38^−/−^ + GFP pair; *P* = 0.3698 compared with the CD38^−/−^ + OT/CD38^−/−^ + GFP pair; *P* = 1.00 compared with the CD38^*−/−*^ + OT/CD38^*−/−*^ + HCD38 pair). This frequency of retrieval is 100% of the frequency shown by the wild-type sires.

**Figure 1:**
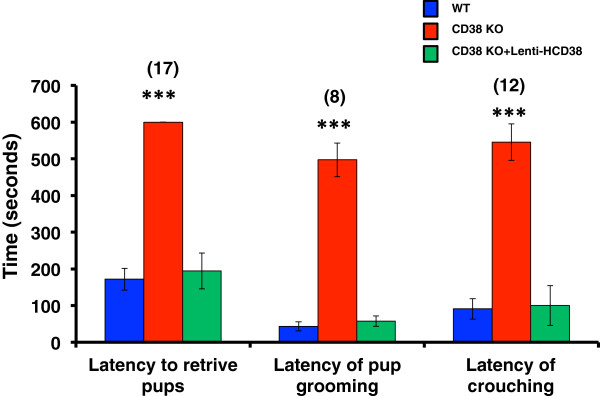
**Latencies in retrieval, pup grooming, and crouching by sires.** Times to the beginning of retrieval (left), grooming (center), and crouching (right) by sires in wild-type (blue) and CD38 knockout sires with (green) or without (red) re-expression of CD38 in the NAcc. Number of experiments is shown in parentheses. One-way ANOVA followed by Bonferroni’s *post hoc* test: *F*_2,66_ = 36.81 (*P* = 0.0000) for retrieval, *F*_2,24_ = 112.59 (*P* = 0.0000) for grooming and *F*_2,23_ = 12.23 (*P* =0.0002) for crouching, respectively. ***P = 0.0000 (two-tailed *t*-test) between groups of CD38 knockout mice and wild-type or CD38 knockout with HCD38 re-expression.

Different parameters and other parental behaviors were additionally observed to examine the effects of HCD38 re-expression in the NAcc in CD38^−/−^ sires. The latency of onset of retrieval in CD38^−/−^ sires, which was significantly greater (slower) than that in the wild-type mice, reverted to a wild-type value in the CD38^−/−^ sires expressing HCD38 (Figure 1, left; n = 17, *P* = 0.0000, One-way ANOVA). Latencies of onset of pup grooming (Figure 1, center) and crouching (Figure 1, right), which were significantly greater (slower) in CD38^−/−^ sires than those of wild-type males, reverted to wild-type values in the CD38^−/−^ sires expressing HCD38 (n = 8 or 12; *P* = 0.0000 and 0.0002, respectively; One-way ANOVA).

**Figure 2:**
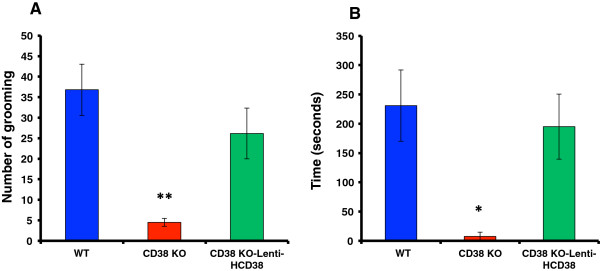
**Grooming and crouching by sires.** Frequency of grooming **(A)** and duration of crouching **(B)** in wild-type (blue) and CD38 knockout sires with (green) or without (red) re-expression of CD38 in the NAcc. N = 10 males in each group. A One-way ANOVA was used followed by Bonferroni’s *post hoc* test: *F*_2,24_ = 6.05 (*P* = 0.0075) for grooming and *F*_2,24_ = 3.20 (*P* = 0.0584) for crouching. **P < 0.01 and *P < 0.05, two-tailed *t*-tests, respectively.

The frequency of pup grooming was less (Figure 2A) and the duration of crouching was shorter (Figure 2B) in CD38^−/−^ sires (n = 8 and 12; **P < 0.01 and *P < 0.05, respectively). These behaviors reverted to wild-type values by expressing of HCD38 in the NAcc in CD38^−/−^ sires (n = 10; *P* = 0.0075 and 0.0584, respectively; One-way ANOVA).

**Figure 3:**
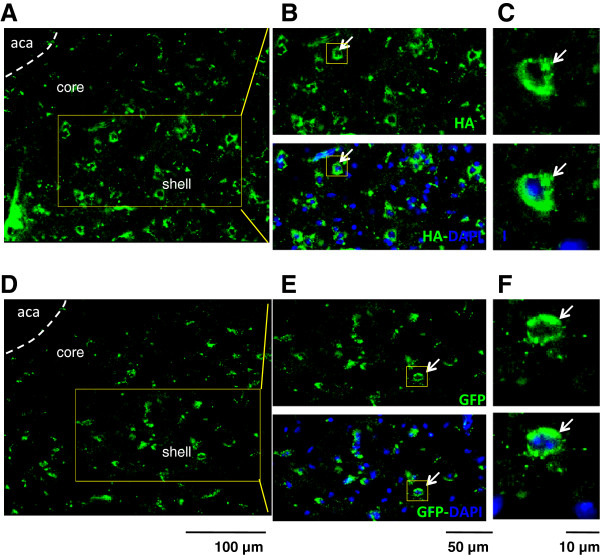
**Representative fluorescence imaging of CD38 and DAPI in the NAcc.** CD38^−/−^ sires were infected with either lenti-HA-HCD38 **(A-C)** or GFP **(D-F)**. CD38 was stained using an anti-HA antibody. Images are enlarged in **B** and **C** or **E** and **F** as indicated by arrows. Scales are 100 μm (for **A** and **D**), 50 μm (for **B** and **E**) and 10 μm (for **C** and **F**), respectively. Aca: anterior commissure anterior; core: NAcc core; shell: NAcc shell.

Finally, we confirmed the re-expression of HCD38 in the NAcc of CD38^−/−^ mice by two histological and functional methods. First, as previously reported [39], fluorescence images in the hypothalamus showed the expression of HA-tagged HCD38 (Figure 3A-C) in discrete cells in the NAcc. GFP was used as the expression control in the same area (Figure 3D-F).

**Figure 4:**
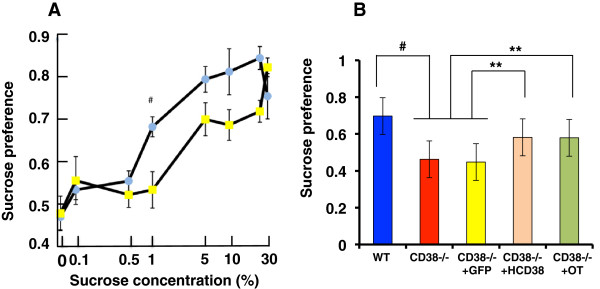
**Effect of CD38 re-expression in the NAcc on sucrose preference in wild-type and knockout mice. (A)** Sucrose uptake ratio (preference) by CD38^*+/+*^ (blue circles) and CD38^*−/−*^ (yellow circles) young adult (non-parental) males at the indicated concentrations of sucrose over water in the two-bottle test over 24 hours. N = 20 each out of a total of 320 males. A Two-way ANOVA was used: wild-type mice (*F*_5,124_ = 12.97, *P* = 0.000), CD38^−/−^ mice (*F*_5,124_ = 16.97, *P* = 0.0000) and their interaction with sucrose concentrations (*F*_5,124_ = 3.80, *P* = 0.0006) were found to be significant factors. A two-tailed Student *t*-test was used to test significance of the difference between CD38^*+/+*^ and CD38^*−/−*^ mice at 1%, ^*#*^*P* < 0.002. **(B)** Sucrose preference tested with 1% sucrose in CD38^*+/+*^ (blue) and CD38^*−/−*^ mice treated with (green) or without (red) OT and CD38^*−/−*^ mice expressing either GFP (yellow) or HCD38 (amber). N = 7 males in each group. A One-way ANOVA followed by Bonferroni’s *post hoc* test was performed: *F*_4,88_ =11.00, *P* = 0.0000. A two-tailed Student *t*-test was performed: ^*#*^*P* = 0.000, **P < 0.05.

Second, we examined sucrose preference to quantify dysfunction in the sensory system and/or adhedonic effects as a component of the NAcc functions in CD38^−/−^ mice. We used young adult males instead of sires in these experiments, because many mice (approximately 320) were required (Figure 4A). CD38^−/−^ males exhibited a significantly lower dose-dependent sucrose preference, as shown by the right shift in drinking 0.1% - 30% sucrose solutions compared with water using the two-bottle method (50, 51; Figure 4A). A Two-way ANOVA revealed that wild-type mice (*F*_5,124_ = 12.97, *P* = 0.0000), CD38^−/−^ mice (*F*_5,124_ = 16.97, *P* = 0.0000) and their interaction with sucrose concentrations (*F*_5,124_ = 3.80, *P* = 0.0006) were significant effects.

The lower preference ratio at 1% sucrose in CD38^−/−^ males was not reversed by GFP re-expression (Figure 4B). The preference was reversed by expression of HCD38 (*P* < 0.05). No additive effect was obtained by the re-expression of HCD38 and treatment with OT (One-way ANOVA, *F*_4,48_ = 11.00, *P* = 0.0000), most likely because of a ceiling effect (Figure 4B).

## Discussion

The current results demonstrate for the first time that CD38-deficient sires display no parental behavior. This finding is consistent with the previous observation that CD38-deficient knockout dams displayed impaired parental behavior under such stressful conditions as the removal of their pups from the nesting arena [39]. CD38 knockout sires and dams show different degrees of recovery after subcutaneous administration of OT (exogenous OT). The frequencies of the behaviors in OT-treated knockout sires recover to approximately 50% of those of wild-type sires with OT-treated dams (Table 1).

Local re-expression of HCD38 in the NAcc caused the frequency of retrieval behavior to recover to approximately 80% in sires. Addition of OT was necessary for full recovery (Table 2). It has been shown that OT is released directly from the recurrent terminals of hypothalamic oxytocinergic neurons to the receptors in the NAcc [24]. This suggests that further calcium signal amplification by CD38 downstream of the OT receptors may be required in the NAcc. This might be explained by the requirement for the OT signal to be received from the mPOA before transmission to the NAcc, as has been previously reported [23,25]. It is therefore likely that even in sires, the expression of CD38 in the NAcc enabled the integration of OT signals from both the mPOA and the hypothalamus and that this integrated signal might be conveyed to the VP, which is the brain region that initiates maternal behavior [1,4,19].

We demonstrated in this study that the sensory system and adhedonic effects of the reward system in the NAcc [58-60] were partially disrupted in CD38 knockout male mice (Figure 4). However, this behavior was reversed by exogenous OT or re-expression of CD38 in the NAcc, clearly indicating the involvement of the oxytocinergic system in the NAcc. We examined sucrose preference by young adult males to show the involvement of CD38 in the NAcc in the reward system. In this case, we thought that it would be better to use adult males with no sexual experience.

The NAcc has been extensively implicated in the regulation of maternal behavior and processing of pup-related stimuli relevant to maternal behavior: c-Fos expression is increased in the NAcc in maternally behaving rats [61-63]; maternal interaction is associated with dopamine release in the NAcc [64,65]; microinjection of dopamine receptor antagonists into the NAcc of female rats disrupts maternal behavior [66-68]; cycloheximide, a protein synthesis inhibitor, injected into the shell of the NAcc disrupts maternal behavior in rats [69]; and electrolytic- and 6-hydroxydopamine-induced lesions of the NAcc disrupt maternal behavior in rats [70-72]. Therefore, the neural circuitry comprising the NAcc, the amygdala, the ventral prefrontal cortex and the anterior cingulate cortex is thought to be involved in cue-evoked (including cues produced by pups) reward-seeking behavior [72].

In relation to the efficacy of OT in CD38 knockout mice, it has been reported that OT receptors are found in the mesocorticolimbic system and that stimulation of OT receptors in the VTA and NAcc areas influences motivated behavior; stimulation of OT receptors in the NAcc facilitates “spontaneous” maternal behavior in adult female prairie voles [24]; and OT receptors in the NAcc shell are involved in the consolidation of maternal memory in postpartum rats [26].

Although we did not examine the effect, it is known that OT appears to impact dopaminergic activity within the mesocorticolimbic dopamine system, which is crucial not only for reward and motivated behavior but also for the expression of affiliative and abuse behaviors [73]. It has been shown that application of an OT receptor antagonist via intracerebroventricular injection significantly diminishes dopaminergic release by a dopamine agonist in the NAcc and the pro-erectile effect of dopamine [74]. If social deficits associated with autism spectrum disorders (ASDs) are related to reward circuitry dysfunction, it might be expected that patients with ASD sometimes show hypoactivation in the mesocorticolimbic circuitry, including the NAcc, in response to social and non-social reward [75,76].

We have reported that paternal behavior is mediated by USVs and pheromones emitted by the dams [17]. In preliminary experiments, we tested the relationship between CD38 knockouts, USV, and retrieval: CD38^−/−^ dams did not emit 38 kHz USV when paired with either the wild-type or CD38^−/−^ sires. Likewise, CD38^−/−^ males did not show any parental behavior when paired with wild-type 38-kHz USV-emitting dams. These preliminary results suggest that the CD38^−/−^ mice demonstrate no ability to respond to the USVs.

We used human CD38 instead of mouse CD38 in the present experiment to reverse these effects in the NAcc. Previously, we used human CD38 for local re-expression in the hypothalamus because we wanted to compare recovery of social behavior (investigation time of males in response to repeated encounters with female intruders) caused by intact CD38 with arginine at the 140^th^ amino acid or mutant CD38 with tryptophan at the 140^th^ amino acid [39,47]. Full behavioral recovery was observed with human CD38 in CD38 knockout mice [39], although the homology at the amino acid level between human and mouse CD38 is only 59%. We thus used the same lentivirus vector harboring human CD38 in the current experiment.

## Conclusions

The mPOA-VTA-NAcc-VP neurocircuit has been suggested to be involved in maternal behavior by rodent dams. In this study, we propose that the NAcc in this neurocircuit is shared between sires and dams in mice. CD38 in the NAcc and OT are the required components for the expression of paternal behavior in male mice.

## Methods

### Animals

Wild-type male and female Slc:ICR mice (Institute of Cancer Research of the Charles River Laboratories, Inc., Wilmington, MA) were obtained from Japan SLC, Inc. (Hamamatsu, Japan) through a local distributor (Sankyo Laboratory Service Corporation, Toyama, Japan). The offspring of the wild-type and CD38^−/−^ mice were born in our laboratory colony. The average litter size of wild-type dams was 13.5 ± 0.67 (n = 30) and 10.3 ± 0.28 (n = 15) in CD38^−/−^ dams (*P* = 0.0013). Although pup number was smaller, it was not extremely low in the knockout dams. Pups were weaned at 21–28 days of age and housed in same-sex groups of 5 animals until pairing. A male and female of each genotype were paired and kept in a nursing cage in our laboratory under standard conditions (24°C; 12-h light/dark cycle, lights on at 08:00) with food and water provided *ad libitum*. All of the animal experiments were performed in accordance with the Fundamental Guidelines for the Proper Conduct of Animal Experiments and Related Activities in Academic Research Institutions under the jurisdiction of the Ministry of Education, Culture, Sports, Science and Technology of Japan and were approved by the Committee on Animal Experimentation of Kanazawa University.

### CD38 knockout mice

The procedure for producing CD38 knockout mice has been described in detail [48]. Briefly, a 15-kbp mouse genomic DNA (*Bam*HI-*Bam*HI) fragment containing the first exon of the CD38 gene was cloned from a TT2 embryonic stem (ES) cell genomic library. The correctly targeted ES cells were injected into eight-cell embryos of ICR mice to produce germ-line chimeras. The mutant and wild-type mice that were used were the littermates intercrossed between male and female heterozygotes that had been backcrossed to ICR mice for over 10 generations.

### Paternal retrieval test

Virgin males and females of identical genotypes were paired at 45–55 days. A single male and a single female were continuously housed together in a standard mouse maternity cage from the mating period to the delivery of the pups and then to postnatal day 3–5. All of the family units consisted of a new sire and dam and their first litter of each genotype and all were experimentally naïve. The sire and dam were placed for 10 min in a clean cage with new woodchip bedding, but the pups were left in the nest in the original cage. Five pups were randomly selected from the litter and placed individually at a site remote from the nest in the original cage. The sires were returned to the original home cage in the presence of their five biological pups to assess parental behavior. Parental retrieval behavior (latency to retrieve the first pup and per cent of sires exhibiting retrieval) was examined for 10 min following the reunion. The behavioral tests were carried out in a randomly mixed sequence of experimental groups. Experiments were usually performed at 10:00–15:00 hours.

We defined retrieval as positive if the sires carried all 5 pups to the original nesting place or within two thirds of the distance between the nest and the place at which the pups had been placed [17]. We also observed other parental behaviors (grooming, crouching and huddling) as defined by Gubernick and Alberts [77]. Animals in this and subsequent experiments were tested only once.

### Viral preparation and lentiviral vector transfection

Vesicular Stomatitis Virus-G protein pseudotyped lentiviral vectors were designed to express either GFP or HA-tagged human CD38 under the control of the murine embryonic stem cell virus promoter. The viral vector particles were produced and titrated as previously described [39]. Lentiviral vectors were injected stereotactically (LR, 1.0 mm, AP 1.18 mm, DV, 4.2 mm) into the NAcc of 6-week-old male CD38^−/−^ mice that had been paired with CD38^−/−^ females for 3 days. After 3–4 weeks of lentiviral vector suspension injection, the animals were used for the experiments on the 3^rd^ to 5^th^ PND after the pair produced pups. Two-μl viral suspensions were injected with a 10-μl Hamilton syringe at a rate of 0.2 μl/min with an automatic injector (Micro4, WPI, Sarasota, FL) and the needle was then left in place for an additional 5–10 min before being withdrawn.

We examined sires that were genetically manipulated with local re-expression of HCD38 because it was difficult to manipulate the pregnant females for local re-expression of HCD38.

### CD38 immunostaining

After the parental behavior test, the mice were immediately anesthetized and perfused intracardially with cold PBS followed by cold 4% paraformaldehyde (PFA) in PBS. Brains were removed and postfixed overnight in a 4% PFA solution at 4°C. Tissue sections were preincubated in blocking solution (3% BSA and 0.3% Triton X-100 in PBS) for 1 h and then incubated for 12 h overnight with anti-HA and anti-GFP antibody (1:100; Sigma mouse monoclonal anti-HA antibody and 1:500 MBL rabbit anti-GFP antibody) in a blocking solution. After three washes with washing buffer, the sections were incubated with goat anti-mouse IgG antibody coupled with Alexa Fluor 488 (Invitrogen, Carlsbad, CA), anti-rabbit IgG antibody coupled with Alexa Fluor 488 (Invitrogen) and DAPI (1:2000 Wako Pure Chemical Industries, Ltd., Osaka, Japan) in a blocking solution for 1 h at room temperature. Images were taken using an Olympus IX71 inverted microscope (Tokyo, Japan) equipped with a cooled CCD camera (Cool SNAP HQ2; Roper Scientific, Tucson, AZ).

### Intake of sucrose solution and water in male mice

Experimentally naïve young adult ICR wild-type and CD38^−/−^ male mice (6–8 weeks old) were given a two-bottle choice between distilled water and different concentrations of sucrose solution, which were both available *ad libitum* in drinking pipettes calibrated at 0.25-ml increments, as previously reported [52,53]. The bottle positions remained constant. Fresh sucrose solution was prepared each day. A 1% concentration was used based on our previous results as well as earlier reports that mice prefer sucrose solution. Cumulative water and sucrose intakes were recorded for 4 or 24 h. Food was provided *ad libitum* but food intake was not recorded in this experiment. The genotype difference in the two-bottle choice between the 1% sucrose solution and water was clear in males. We therefore used male mice for the sucrose preference test. The 1% sucrose preference was measured after an intraperitoneal injection of OT (100 ng/kg body weight) and the lentiviral re-expression of GFP/HCD38 in the nucleus of the CD38^−/−^ male mice.

### Statistical analysis

Fisher’s exact tests and two-tailed Student’s *t* tests were used for single comparisons between two groups. The rest of the data were analyzed using One-way or Two-way analyses of variance (ANOVA) for two or three components. *Post hoc* comparisons were performed only when the main effect showed statistical significance. *P*-values of the multiple comparisons were adjusted using Bonferroni’s correction. All of the analyses were performed using STATA data analysis and statistical software (Stata Corp LP, College Station, TX).

## Competing interests

The authors declare that they have no competing interests.

## Authors’ contributions

SA, NK, JZ, ML, SMC, OL, YKK, TN, AAF, and HHig conducted most of the experiments, and analyzed all the data. TK, YY, ST, and HO made knockout mice. HHir produced lentiviral vectors. HHig designed the study. HHig, ABS and HO wrote the manuscript. All authors read and approved the final manuscript.

## References

[B1] Bridges RS “Neurobiology of the parental Brain” pp 1–550 2008 Burlington, MA, USA: Academic Press

[B2] Douglas AJ Mother-offspring dialogue in early pregnancy: Impact of adverse environment on pregnancy maintenance and neurobiology Prog Neuropsychopharmacol Biol Psychiatry 2011 35 1167 1177 10.1016/j.pnpbp.2010.07.024 20688125

[B3] Bridges RS Long-term effects of pregnancy and parturition upon maternal responsiveness in the rat Physiol Behav 1975 14 245 249 10.1016/0031-9384(75)90028-1 1169782

[B4] Kuroda KO Tachikawa K Yoshida S Tsuneoka Y Numan M Neuromolecular basis of parental behavior in laboratory mice and rats: with special emphasis on technical issues of using mouse genetics Prog Neuropsychopharmacol Biol Psychiatry 2011 35 1205 1231 10.1016/j.pnpbp.2011.02.008 21338647

[B5] Brunton PJ Russell JA The expectant brain: adapting for motherhood Nat Rev Neurosci 2008 9 11 25 18073776 10.1038/nrn2280

[B6] Wright SL Brown RE Maternal behavior, paternal behavior, and pup survival in CD-1 albino mice (Mus musculus) in three different housing conditions J Comp Psychol 2000 114 183 192 10890590 10.1037/0735-7036.114.2.183

[B7] Lopatina O Liu HX Amina S Hashii M Higashida H Oxytocin-induced elevation of ADP-ribosyl cyclase activity, cyclic ADP-ribose or Ca^2+^ concentrations is involved in autoregulation of oxytocin secretion in the hypothalamus and posterior pituitary in male mice Neuropharmacology 2011 58 50 55 19540855 10.1016/j.neuropharm.2009.06.012

[B8] Kentner AC Abizaid A Bielajew C Modeling dad: animal models of paternal behavior Neurosci Biobehav Rev 2010 34 438 451 10.1016/j.neubiorev.2009.08.010 19744516

[B9] Leuner B Glasper ER Gould E Parenting and plasticity Trends Neurosci 2010 33 465 473 10.1016/j.tins.2010.07.003 20832872 PMC3076301

[B10] Lonstein JS de Vries GJ Sex differences in the parental behavior of rodents Neurosci Biobehav Rev 2000 24 669 686 10.1016/S0149-7634(00)00036-1 10940441

[B11] Young LJ Wang Z Insel TR Neuroendocrine bases of monogamy Trends Neurosci 1998 21 71 75 10.1016/S0166-2236(97)01167-3 9498302

[B12] Carter CS Boone EM Pournajafi-Nazarloo H Bales KL Consequences of early experiences and exposure to oxytocin and vasopressin are sexually dimorphic Dev Neurosci 2009 31 332 341 10.1159/000216544 19546570 PMC2820581

[B13] Wynne-Edwards KE Timonin ME Paternal care in rodents: weakening support for hormonal regulation of the transition to behavioral fatherhood in rodent animal models of biparental care Horm Behav 2007 52 114 121 10.1016/j.yhbeh.2007.03.018 17482188

[B14] Gubernick DJ Alberts JR Postpartum maintenance of paternal behaviour in the biparental California mouse, Peromyscus californicus Anim Behav 1989 37 656 664

[B15] de Jong TR Chauke M Harris BN Saltzman W From here to paternity: neural correlates of the onset of paternal behavior in California mice (Peromyscus californicus) Horm Behav 2009 56 220 231 10.1016/j.yhbeh.2009.05.001 19433091

[B16] Rosenblatt JS Nonhormonal basis of maternal behavior in the rat Science 1967 156 1512 1514 10.1126/science.156.3781.1512 5611028

[B17] Liu HX Lopatina O Higashida C Fujimoto H Akther S Inzhutova A Liang M Zhong J Tsuji T Yoshihara T Sumi K Ishiyama M Ma WJ Ozaki M Yagitani S Yokoyama S Mukaida N Sakurai T Hori O Yoshioka K Hirao A Kato Y Ishihara K Kato I Okamoto H Cherepanov SM Salmina AB Hirai H Asano M Brown DA Nagano I Higashida H Displays of paternal mouse pup retrieval following communicative interaction with maternal mates Nat Commun 2013 1346 1 8 10.1038/ncomms2336 PMC4089749 23299896

[B18] Fujimoto H Liu HX Lopatina O Brown DA Higashida H Scopolamine modulates paternal parental retrieval behavior in mice induced by the maternal mate Neurosci Lett 2013 546 63 66 23669641 10.1016/j.neulet.2013.04.059

[B19] Numan M Numan MJ Schwarz JM Neuner CM Flood TF Smith CD Medial preoptic area interactions with the nucleus accumbens-ventral pallidum circuit and maternal behavior in rats Behav Brain Res 2005 158 53 68 10.1016/j.bbr.2004.08.008 15680194

[B20] Stolzenberg DS Numan M Hypothalamic interaction with the mesolimbic DA system in the control of the maternal and sexual behaviors in rats Neurosci Biobehav Rev 2011 35 826 847 10.1016/j.neubiorev.2010.10.003 20955733

[B21] Kuroda KO Li M Fleming AS The nucleus accumbens shell is critical for normal expression of pup-retrieval in postpartum female rats Behav Brain Res 2003 145 99 111 10.1016/S0166-4328(03)00135-9 14529809

[B22] Olazábal D Pereira M Agrati D Ferreira A Fleming AS González-Mariscal G Lévy F Lucion AB Morrell JI Numan M Uriarte N New theoretical and experimental approaches on maternal motivation in mammals Neurosci Biobehav Rev 2013 S0149-7634(13)00099-7 10.1016/j.neubiorev.2013.04.003 23608127

[B23] Ross HE Cole CD Smith Y Neumann ID Landgraf R Murphy AZ Young LJ Characterization of the oxytocin system regulating affiliative behavior in female prairie voles Neuroscience 2009 162 892 903 10.1016/j.neuroscience.2009.05.055 19482070 PMC2744157

[B24] Olazábal DE Young LJ Oxytocin receptors in the nucleus accumbens facilitate "spontaneous" maternal behavior in adult female prairie voles Neuroscience 2006 141 559 568 10.1016/j.neuroscience.2006.04.017 16725274

[B25] Shahrokh DK Zhang TY Diorio J Gratton A Meaney MJ Oxytocin-dopamine interactions mediate variations in maternal behavior in the rat Endocrinology 2010 151 2276 2286 10.1210/en.2009-1271 20228171 PMC2869254

[B26] D'Cunha TM King SJ Fleming AS Lévy F Oxytocin receptors in the nucleus accumbens shell are involved in the consolidation of maternal memory in postpartum rats Horm Behav 2011 59 14 21 10.1016/j.yhbeh.2010.09.007 20932839

[B27] Castel M Gainer H Dellmann HD Neuronal secretory systems Int Rev Cytol 1984 88 303 459 6203862 10.1016/s0074-7696(08)62760-6

[B28] Insel TR The challenge of translation in social neuroscience: a review of oxytocin, vasopressin, and affiliative behavior Neuron 2010 65 768 779 10.1016/j.neuron.2010.03.005 20346754 PMC2847497

[B29] Neumann ID Landgraf R Balance of brain oxytocin and vasopressin: implications for anxiety, depression, and social behaviors Trends Neurosci 2012 35 649 659 10.1016/j.tins.2012.08.004 22974560

[B30] Hashimoto H Uezono Y Ueta Y Pathophysiological function of oxytocin secreted by neuropeptides: A mini review Pathophysiol 2012 19 283 298 10.1016/j.pathophys.2012.07.005 22902166

[B31] Higashida H Yokoyama S Kikuchi M Munesue T CD38 and its role in oxytocin secretion and social behavior Horm Behav 2012 61 351 358 10.1016/j.yhbeh.2011.12.011 22227279

[B32] Higashida H Yokoyama S Huang JJ Liu L Ma WJ Akther S Higashida C Kikuchi M Minabe Y Munesue T Social memory, amnesia, and autism: brain oxytocin secretion is regulated by NAD^+^ metabolites and single nucleotide polymorphisms of CD38 Neurochem Int 2012 61 828 838 10.1016/j.neuint.2012.01.030 22366648

[B33] Kumsta R Heinrichs M Oxytocin, stress and social behavior: neurogenetics of the human oxytocin system Curr Opin Neurobiol 2013 23 11 16 10.1016/j.conb.2012.09.004 23040540

[B34] Modi ME Young LJ The oxytocin system in drug discovery for autism: animal models and novel therapeutic strategies Horm Behav 2012 61 340 350 10.1016/j.yhbeh.2011.12.010 22206823 PMC3483080

[B35] Yamasue H Yee JR Hurlemann R Rilling JK Chen FS Meyer-Lindenberg A Tost H Integrative approaches utilizing oxytocin to enhance prosocial behavior: from animal and human social behavior to autistic social dysfunction J Neurosci 2012 32 14109 14117 10.1523/JNEUROSCI.3327-12.2012 23055480 PMC6622380

[B36] Okamoto H Takasawa S Recent advances in the Okamoto model: the CD38-cyclic ADP-ribose signal system and the regenerating gene protein (Reg)-Reg receptor system in beta-cells Diabetes 2002 51 Suppl 3 S462 S473 12475791 10.2337/diabetes.51.2007.s462

[B37] Malavasi F Deaglio S Funaro A Ferrero E Horenstein AL Ortolan E Vaisitti T Aydin S CD38 Evolution and function of the ADP ribosyl cyclase/CD38 gene family in physiology and pathology Physiol Rev 2008 88 841 888 10.1152/physrev.00035.2007 18626062

[B38] Lee HC The cyclic ADP-ribose/NAADP/CD38-signaling pathway: Past and present Messenger 2012 1 16 33 10.1166/msr.2012.1005

[B39] Jin D Liu HX Hirai H Torashima T Nagai T Lopatina O Shnayder NA Yamada K Noda M Seike T Fujita K Takasawa S Yokoyama S Koizumi K Shiraishi Y Tanaka S Hashii M Yoshihara T Higashida K Islam MS Yamada N Hayashi K Noguchi N Kato I Okamoto H Matsushima A Salmina A Munesue T Shimizu N Mochida S Asano M Higashida H CD38 is critical for social behaviour by regulating oxytocin secretion Nature 2007 446 41 45 10.1038/nature05526 17287729

[B40] Munesue T Yokoyama S Nakamura K Anitha A Yamada K Hayashi K Asaka T Liu HX Jin D Koizumi K Islam MS Huang JJ Ma WJ Kim UH Kim SJ Park K Kim D Kikuchi M Ono Y Nakatani H Suda S Miyachi T Hirai H Salmina A Pichugina YA Soumarokov AA Takei N Mori N Tsujii M Sugiyama T Yagi K Yamagishi M Sasaki T Yamasue H Kato N Hashimoto R Taniike M Hayashi Y Hamada J Suzuki S Ooi A Noda M Kamiyama Y Kido MA Lopatina O Hashii M Amina S Malavasi F Huang EJ Zhang J Shimizu N Yoshikawa T Matsushima A Minabe Y Higashida H Two genetic variants of CD38 in subjects with autism spectrum disorder and controls Neurosci Res 2010 67 181 191 10.1016/j.neures.2010.03.004 20435366

[B41] Lerer E Levi S Israel S Yaari M Nemanov L Mankuta D Nurit Y Ebstein RP Low CD38 expression in lymphoblastoid cells and haplotypes are both associated with autism in a family-based study Autism Res 2010 3 293 302 10.1002/aur.156 21182206

[B42] Feldman R Gordon I Influs M Gutbir T Ebstein RP Parental oxytocin and early caregiving jointly shape children's oxytocin response and social reciprocity Neuropsychopharmacology 2013 38 1154 1162 10.1038/npp.2013.22 23325323 PMC3656367

[B43] Feldman R Zagoory-Sharon O Weisman O Schneiderman I Gordon I Maoz R Shalev I Ebstein RP Sensitive parenting is associated with plasma oxytocin and polymorphisms in the OXTR and CD38 genes Biol Psychiatry 2012 72 175 181 10.1016/j.biopsych.2011.12.025 22336563

[B44] Riebold M Mankuta D Lerer E Israel S Zhong S Nemanov L Monakhov MV Levi S Yirmiya N Yaari M Malavasi F Ebstein RP All-trans retinoic acid upregulates reduced CD38 transcription in lymphoblastoid cell lines from Autism spectrum disorder Mol Med 2011 17 799 806 21528155 10.2119/molmed.2011.00080 PMC3146614

[B45] Sauer C Montag C Wörner C Kirsch P Reuter M Effects of a common variant in the CD38 gene on social processing in an oxytocin challenge study: possible links to autism Neuropsychopharmacology 2012 37 1474 1482 10.1038/npp.2011.333 22278094 PMC3327852

[B46] Nata K Takamura T Karasawa T Kumagai T Hashioka W Tohgo A Yonekura H Takasawa S Nakamura S Okamoto H Human gene encoding CD38 (ADP-ribosyl cyclase/cyclic ADP-ribose hydrolase): organization, nucleotide sequence and alternative splicing Gene 1997 186 285 292 10.1016/S0378-1119(96)00723-8 9074508

[B47] Yagui K Shimada F Mimura M Hashimoto N Suzuki Y Tokuyama Y Nata K Tohgo A Ikehata F Takasawa S Okamoto H Makino H Saito Y Kanatsuka A A missense mutation in the CD38 gene, a novel factor for insulin secretion: association with Type II diabetes mellitus in Japanese subjects and evidence of abnormal function when expressed in vitro Diabetologia 1998 41 1024 1028 10.1007/s001250051026 9754820

[B48] Kato I Yamamoto Y Fujimura M Noguchi N Takasawa S Okamoto H CD38 disruption impairs glucose-induced increases in cyclic ADP-ribose, [Ca^2+^]_i_, and insulin secretion J Biol Chem 1999 274 1869 1872 10.1074/jbc.274.4.1869 9890936

[B49] Mirenowicz J Schultz W Preferential activation of midbrain dopamine neurons by appetitive rather than aversive stimuli Nature 1996 379 449 451 10.1038/379449a0 8559249

[B50] Numan M Motivational systems and the neural circuitry of maternal behavior in the rat Dev Psychobiol 2007 49 12 21 10.1002/dev.20198 17186513

[B51] Everitt BJ Parkinson JA Olmstead MC Arroyo M Robledo P Robbins TW Associative processes in addiction and reward. The role of amygdala-ventral striatal subsystems Ann NY Acad Sci 1999 877 412 438 10.1111/j.1749-6632.1999.tb09280.x 10415662

[B52] Amico JA Vollmer RR Cai HM Miedlar JA Rinaman L Enhanced initial and sustained intake of sucrose solution in mice with an oxytocin gene deletion Am J Physiol Regul Integr Comp Physiol 2005 289 R1798 R1806 10.1152/ajpregu.00558.2005 16150836

[B53] Miedlar JA Rinaman L Vollmer RR Amico JA Oxytocin gene deletion mice overconsume palatable sucrose solution but not palatable lipid emulsions Am J Physiol Regul Integr Comp Physiol 2007 293 R1063 R1068 10.1152/ajpregu.00228.2007 17596329

[B54] Chaudhury D Walsh JJ Friedman AK Juarez B Ku SM Koo JW Ferguson D Tsai HC Pomeranz L Christoffel DJ Nectow AR Ekstrand M Domingos A Mazei-Robison MS Mouzon E Lobo MK Neve RL Friedman JM Russo SJ Deisseroth K Nestler EJ Han MH Rapid regulation of depression-related behaviours by control of midbrain dopamine neurons Nature 2013 493 532 536 23235832 10.1038/nature11713 PMC3554860

[B55] Martínez-Hernández J Lanuza E Martínez-García F Lesions of the dopaminergic innervation of the nucleus accumbens medial shell delay the generation of preference for sucrose, but not of sexual pheromones Behav Brain Res 2012 226 538 547 10.1016/j.bbr.2011.10.013 22019343

[B56] de Araujo IE Oliveira-Maia AJ Sotnikova TD Gainetdinov RR Caron MG Nicolelis MA Simon SA Food reward in the absence of taste receptor signaling Neuron 2008 57 930 941 10.1016/j.neuron.2008.01.032 18367093

[B57] Stolzenberg DS McKenna JB Keough S Hancock R Numan MJ Numan M Dopamine D(1) receptor activation of adenylyl cyclase, not phospholipase C, in the nucleus accumbens promotes maternal behavior onset in rats Horm Behav 2010 57 96 104 10.1016/j.yhbeh.2009.09.014 19799904

[B58] McCutcheon JE Beeler JA Roitman MF Sucrose-predictive cues evoke greater phasic dopamine release than saccharin-predictive cues Synapse 2012 66 346 351 10.1002/syn.21519 22170625 PMC3269555

[B59] Perry ML Baldo BA Andrzejewski ME Kelley AE Muscarinic receptor antagonism causes a functional alteration in nucleus accumbens μ-opiate-mediated feeding behavior Neuropharmacology 2013 67 521 531 18761381 10.1016/j.bbr.2008.08.002 PMC2657318

[B60] Avena NM Rada PV Cholinergic modulation of food and drug satiety and withdrawal Physio Behav 2012 106 332 336 10.1016/j.physbeh.2012.03.020 PMC4361033 22465312

[B61] Fleming AS Suh EJ Korsmit M Activation of Fos-like immunoreactivity in the medial preoptic area and limbic structures of maternal motivation and social interactions in rats Behav Neurosci 1994 108 724 734 7986366 10.1037//0735-7044.108.4.724

[B62] Stack EC Balakrishnan R Numan MJ Numan M A functional neuroanatomical investigation of the role of the medial preoptic area in neural circuits regulating maternal behaviour Behav Brain Res 2002 131 17 36 10.1016/S0166-4328(01)00370-9 11844569

[B63] Lonstein JS Simmons DA Swann JM Stern JM Forebrain expression of c-fos due to active maternal behaviour in lactating rats Neuroscience 1998 82 267 281 9483519 10.1016/s0306-4522(97)00283-2

[B64] Champagne FA Chretien P Stevenson CW Zhang TY Gratton A Meaney MJ Variations in nucleus accumbens dopamine is associated with individual differences in maternal behavior in the rat J Neurosci 2004 24 4114 4123 10.1523/JNEUROSCI.5322-03.2004 PMC6729285 15115806

[B65] Hansen S Bergvall AH Nyiredi S Interaction with pups enhances dopamine release in the ventral striatum of maternal rats: a microdialysis study Pharmacol Biochem Behav 1993 45 673 676 10.1016/0091-3057(93)90523-V 7687357

[B66] Keer SE Stern JM Dopamine receptor blockade in the nucleus accumbens inhibits maternal retrieval and licking, but enhances nursing behaviour in lactating rats Physiol Behav 1999 67 659 669 10.1016/S0031-9384(99)00116-X 10604835

[B67] Numan M Numan MJ Pliakou N Stolzenberg DS Mullins OJ Murphy JM Smith CD The effects of D1 or D2 dopamine receptor antagonism in the medial preoptic area, ventral pallidum, or nucleus accumbens on the maternal retrieval response and other aspects of maternal behavior in rats Behav Neurosci 2005 119 1588 1604 16420162 10.1037/0735-7044.119.6.1588

[B68] Silva MR Bernardi MM Cruz-Casallas PE Felicio LF Pimozide injections into nucleus accumbens disrupt maternal behaviour in lactating rats Pharmacol Toxicol 2003 93 42 47 10.1034/j.1600-0773.2003.930106.x 12828573

[B69] Li M Fleming AS Differential involvement of the nucleus accumbens shell and core subregions in maternal memory in postpartum female rats Behav Neurosci 2003 117 426 445 12802872 10.1037/0735-7044.117.3.426

[B70] Hansen S Maternal behavior of female rats with 6-OHDA lesions in the ventral striatum: characterization of the pup retrieval deficit Physiol Behav 1994 55 615 620 10.1016/0031-9384(94)90034-5 8190785

[B71] Li M Fleming AS The nucleus accumbens shell is critical for normal expression of pup-retrieval in postpartum female rats Behav Brain Res 2003 145 99 111 10.1016/S0166-4328(03)00135-9 14529809

[B72] Sesack SR Grace AA Cortico-basal ganglia reward network: microcircuitry Neuropsychopharmacology 2010 35 27 47 10.1038/npp.2009.93 19675534 PMC2879005

[B73] Young EA Dreumont SE Cunningham CL Role of nucleus accumbens dopamine receptor subtypes in the learning and expression of alcohol-seeking behavior Neurobiol Learn Mem 2013 doi: S1074-7427(13)00085-3 10.1016/j.nlm.2013.05.004 PMC3842358 23742917

[B74] Succu S Sanna F Melis T Boi A Argiolas A Melis MR Stimulation of dopamine receptors in the paraventricular nucleus of the hypothalamus of male rats induces penile erection and increases extra-cellular dopamine in the nucleus accumbens: involvement of central oxytocin Neuropharmacology 2007 52 1034 1043 10.1016/j.neuropharm.2006.10.019 17164075

[B75] Dawson G Webb SJ McPartland J Understanding the nature of face processing impairment in autism: insights from behavioral and electrophysiological studies Dev Neuropsychol 2005 27 403 424 10.1207/s15326942dn2703_6 15843104

[B76] Schultz RT Developmental deficits in social perception in autism: the role of the amygdala and fusiform face area Int J Dev Neurosci 2005 23 125 141 10.1016/j.ijdevneu.2004.12.012 15749240

[B77] Gubernick DJ Alberts JR The biparental care system of the California mouse, Peromyscus californicus J Comp Psychol 1987 101 169 177 3608423

